# Defective hippocampal neurogenesis underlies cognitive impairment by carotid stenosis-induced cerebral hypoperfusion in mice

**DOI:** 10.3389/fncel.2023.1219847

**Published:** 2023-08-11

**Authors:** Enrique Fraga, Violeta Medina, María Isabel Cuartero, Alicia García-Culebras, Isabel Bravo-Ferrer, Macarena Hernández-Jiménez, Juan Manuel Garcia-Segura, Olivia Hurtado, Jesus Miguel Pradillo, Ignacio Lizasoain, María Ángeles Moro

**Affiliations:** ^1^Neurovascular Pathophysiology Group, Cardiovascular Risk Factor and Brain Function Programme, Centro Nacional de Investigaciones Cardiovasculares (CNIC), Madrid, Spain; ^2^Unidad de Investigación Neurovascular, Departamento de Farmacología, Facultad de Medicina, Universidad Complutense de Madrid (UCM), Madrid, Spain; ^3^Instituto de Investigación Hospital 12 de Octubre (i+12), Madrid, Spain; ^4^Instituto Universitario de Investigación en Neuroquímica, Universidad Complutense de Madrid (UCM), Madrid, Spain; ^5^Departamento de Biología Celular, Facultad de Medicina, Universidad Complutense de Madrid (UCM), Madrid, Spain; ^6^ICTS Bioimagen Complutense, Universidad Complutense de Madrid (UCM), Madrid, Spain

**Keywords:** hypoperfusion, carotid stenosis, neurogenesis, hippocampus, vascular cognitive impairment, dementia, aberrant neurogenesis, maladaptive remodeling

## Abstract

Chronic cerebral hypoperfusion due to carotid artery stenosis is a major cause of vascular cognitive impairment and dementia (VCID). Bilateral carotid artery stenosis (BCAS) in rodents is a well-established model of VCID where most studies have focused on white matter pathology and subsequent cognitive deficit. Therefore, our aim was to study the implication of adult hippocampal neurogenesis in hypoperfusion-induced VCID in mice, and its relationship with cognitive hippocampal deficits. Mice were subjected to BCAS; 1 and 3 months later, hippocampal memory and neurogenesis/cell death were assessed, respectively, by the novel object location (NOL) and spontaneous alternation performance (SAP) tests and by immunohistology. Hypoperfusion was assessed by arterial spin labeling-magnetic resonance imaging (ASL-MRI). Hypoperfused mice displayed spatial memory deficits with decreased NOL recognition index. Along with the cognitive deficit, a reduced number of newborn neurons and their aberrant morphology indicated a remarkable impairment of the hippocampal neurogenesis. Both increased cell death in the subgranular zone (SGZ) and reduced neuroblast proliferation rate may account for newborn neurons number reduction. Our data demonstrate quantitative and qualitative impairment of adult hippocampal neurogenesis disturbances associated with cerebral hypoperfusion-cognitive deficits in mice. These findings pave the way for novel diagnostic and therapeutic targets for VCID.

## Introduction

Cognitive impairment of vascular etiology is the second leading cause of dementia, just behind Alzheimer’s disease (AD). VCID, a wide term proposed to encompass the whole range of cognitive dysfunctions of vascular origin, affects millions of people worldwide, with numbers growing as the median age in the western population continues to rise. Chronic cerebral hypoperfusion, due largely to carotid artery stenosis, is one of the main causes of VCID in humans (Iadecola, 2013; Iadecola et al., 2019). Therefore, BCAS in rodents is a commonly used model of cerebral hypoperfusion that recapitulates cognitive decline and white matter lesions found in VCID patients (Shibata et al., 2004; Berry et al., 2019). In this context, BCAS has been shown to lead to inflammation (Duan et al., 2009), oxidative stress (Miyamoto et al., 2013), white matter injury (Coltman et al., 2011), blood-brain barrier disruption (Seo et al., 2013), reduction in oligodendrocytes numbers (Fujita et al., 2010; Ohtomo and Arai, 2020), and working memory impairment in the 8-arm test (Shibata et al., 2007; Coltman et al., 2011) after 1–2 month of hypoperfusion in mice.

While most studies on this VCID model have primarily focused on white matter pathology, some have also reported effects on gray matter regions, such as the hippocampus (Roberts et al., 2018), including astrocytic changes in the hippocampus, particularly in CA1, and neurodegeneration and cell death (Stevenson et al., 2020; Liu et al., 2021; Shen et al., 2021; Li et al., 2023). However, these studies have not delved deeply into hippocampal processes involved in memory encoding and retrieval. In this context, the hippocampal subgranular zone (SGZ), one of the two adult neurogenic areas, gives rise throughout life to granule neurons that migrate into the granular layer (GL) of the dentate gyrus (DG) where they integrate functionally into the hippocampal circuits. Adult hippocampal neurogenesis (AHN) is thus considered a critical process for the formation and retrieval of spatial memories (Zhao et al., 2008; Cuartero et al., 2021; Li and Guo, 2021). Alterations in hippocampal neurogenesis have been proposed to contribute to the pathogenesis of neurodegenerative diseases such as AD, where decreases in newborn hippocampal neurons were reported in rodents and humans (Donovan et al., 2006; Moreno-Jiménez et al., 2019; Tobin et al., 2019), and post-stroke cognitive impairment (Woitke et al., 2017; Cuartero et al., 2019), supporting the role of AHN in memory deficits. To study their possible contribution in chronic BCAS-induced hypoperfusion, our aim was to explore AHN and hippocampus-dependent cognitive function along with other hippocampal alterations, in a mouse BCAS model. Furthermore, we examined cerebral hypoperfusion at times longer than normally reported, in an attempt to recapitulate the progressive and chronic development of human VCID. Here, our data show that BCAS produces an impairment in both spatial working memory and reference memory in mice after a 3-month period of stenosis. Importantly, concomitant to the cognitive deterioration induced by BCAS, we also found alterations in AHN, in terms of numbers and morphology of newborn neurons, which may result from increased cell death in the DG and/or reduced neuroblast proliferation in the granular layer.

## Materials and methods

All procedures were in accordance with the guidelines of the Animal Welfare Committee of the Centro Nacional de Investigaciones Cardiovasculares Carlos III (CNIC) and the Consejería de Medio Ambiente y Ordenación del Territorio de la Comunidad de Madrid (RD 53/2013; PROEX 047/16, PROEX 193.1/21) following the European directives 86/609/CEE and 2010/63/EU, and are reported according to ARRIVE guidelines. The data that support the findings of this study are available from the corresponding authors upon reasonable request.

### Animals

**FIGURE 1:**
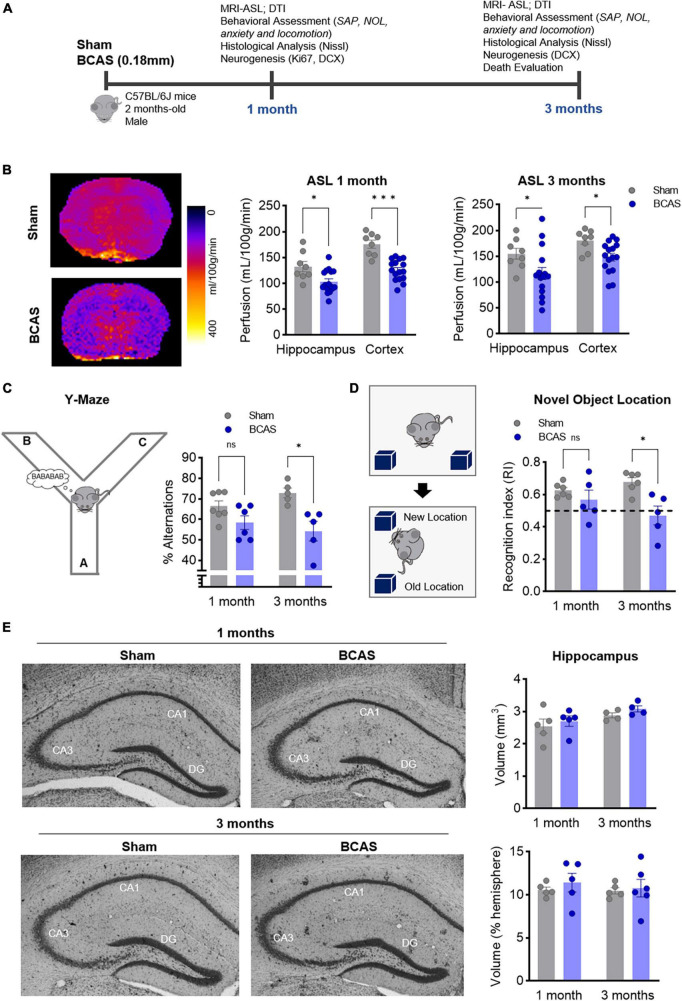
Chronic cerebral hypoperfusion induces hippocampus-memory impairment in mice without signs of hippocampal atrophy. **(A)** Experimental design. **(B)** Arterial spin labeling (ASL) was used to measure CBF after 1 (middle) or 3 months (right) of BCAS in hippocampus and cortex. Representative pictures 3 months after surgery are shown (left) (*n* = 8, 16). **(C)** Percentage of spontaneous alternation in the Y maze at 1–3 months of BCAS (1 month, *n* = 6–7, 3 months, *n* = 5). **(D)** NOL test results expressed as recognition index (*n* = 5). **(E)** Volumetric assessment of hippocampus, normalized by hemisphere volume (*n* = 4–5). Representative Nissl-stained images. Data are mean ± SEM. Statistical assessment by ANOVA and multiple Mann-Whitney tests with Holm-Sidak correction for the paired comparisons. *p < 0.05, ***p < 0.001.

All experiments were performed in male C57BL/6 mice, kept in ventilated cages at 22°C under a 12 h light/12 h dark schedule, with water and chow available *ad libitum*. Seven-10-week-old mice were randomly assigned to sham or BCAS procedure, which was performed with external 0.18 mm microcoils as described (Shibata et al., 2004). Behavioral tests and tissue collection were performed 1 or 3 months after BCAS. The experimental design is shown in Figure 1A.

The calculation of the estimated sample size for each experimental group was performed with the function pwrss.np.2groups() from the {pwrss} package in R. This function allows to obtain an estimated n given the power of the test (β) (80%), significance level (α) (0.05), estimations of the means and standard deviations (SD) from the populations and kappa (relation between the n of both populations). See the details about groups sample size in Supplementary Table 2 and about animals’ allocation in Supplementary Figure 4.

To get the estimations of means and SD, we used the previous experience of our group in the different techniques and assessments when available, as well as in the previously published literatures. Regarding mean and SD of the BCAS population, we assumed and expected a 20% change in respect to the control group when no evidence was available and SD was assumed to be same as control.

Allocation of animals to the different experimental groups was made using the standard = RAND() function in Microsoft Excel. To produce the randomization plan, blocks of X subjects were randomized to the two experimental groups by surgical procedure, sham and BCAS, at each time point (2 experimental groups × X subjects).

The following mice were excluded of further analysis: (a) mice which after the procedure had signs of vague nerve affection (difficulties to breath, movements in circles) or (b) animals which during the experiments showed signs of seriously compromised health status. In both cases animals were euthanized and excluded of any experiment. Moreover, some mice were excluded from some particular experiments if they fitted outlier criteria by Grubb’s method (alpha = 0.05 if only one value was extreme), ROUT (*Q* = 1%) or based in interquartile range (IQR) (excluding values < or > 1.5 IQR, only with *n* = 3–5). The total number of animals used for this project was 183, where 13 of them were excluded due to criteria a or b.

For each animal, two different investigators were involved as follows: a first investigator performed the randomization table. This investigator was the only person aware of the experimental group allocation. A second investigator was responsible for performing the surgical procedure. Finally, a third investigator (also unaware of groups) assessed the behavioral testing and histological procedures.

### Surgery

Seven-10-week-old male mice were randomly assigned to sham or bilateral common carotid artery stenosis (BCAS) procedure. Random numbers were generated using the standard = RAND() function in Microsoft Excel. BCAS was performed using external 0.18 mm microcoils as previously described (Shibata et al., 2004). Briefly, mice were anesthetized and maintained at 2% isoflurane in a mixture of 0.2/0.8 L/min O_2_/air and temperature was kept at 36.5–37°C using a heating blanket. Through a midline cervical incision right common carotid artery was exposed and freed from its sheath. The artery was gently lifted with the help of one 6–0 silk suture and a 0.18 mm microcoil was twined around the artery. After 30 min the same procedure was performed on the left common carotid artery. Animals in the sham group underwent the same procedure without implanting the microcoil.

### Behavioral testing

#### Novel object location (NOL)

A squared experimental box (65 cm × 65 cm × 45 cm) and two identical objects were used. The test consists of two consecutive habituation days where mice were placed in the box with no objects for a 30-min period, followed by the testing day. On testing day, mice were first placed in the box with both objects located at the same side of the box for 5 min. Mice were returned to their home cages for 30 min and then tested again for 5 min but with one of the objects placed in a new location. Behavior was recorded by overhead cameras and exploration time was measured manually for both objects. Recognition index (RI) was expressed as follows:


$$\text{RI}=\left[{\text{time}}_{\mathrm{new}\,\mathrm{locationl}}/\left({\text{time}}_{\mathrm{new}\,\mathrm{location}}+{\text{time}}_{\mathrm{old}\,\mathrm{location}}\right)\right].$$

Exploration of an object was considered when the animal was directly interacting or looking at the object within a 2-cm distance. Climbing on top of the object was not considered as exploration.

#### Y-maze

The Y-Maze consisted of three equal arms (35 cm × 7 cm × 5 cm) separated in 120 degrees angles forming a Y-shaped apparatus. Mice were left free to explore the maze for 5 min, and the total number and sequence of arm entries was notated. Alternation behavior was considered when mice entered all three of the arms consecutively (ABC, BCA, CAB, etc.). Number of alternations was divided by the maximum alternation (total number of entries minus 2) and multiplied by 100 to obtain the spontaneous alternation percentage.

For every behavioral test, testing chambers were rinsed with 70% ethanol between animals to avoid any odor cues. To avoid possible interference due to the manipulation, training, and testing, different mouse cohorts for each set of experiments and tests were used.

#### Forced swimming test

To examine the depressive state Porsolt forced swim test was performed. The apparatus consisted of one plexiglass cylinder (44 cm height × 24 cm diameter). The cylinder was filled with water (23°C), up to a height of 26 cm. Mice were placed into the cylinders, and their behavior was recorded over a 6-min test period. For each mouse the immobile time in the last 5 min was registered.

#### Open field

Each animal was placed in the center of the open field apparatus (plexiglass, 40 cm × 40 cm × 70 cm) and the behavior was recorded for 8 min. Then, the test was analyzed using Ethowatcher. Total distance, time in center and time in the periphery were obtained. The inner region of 40% of the apparatus was considered the center.

#### Swimming motor test

For this test, swimming speed is registered along a thin rectangular pool. It was done similarly as previously described in Carter et al. (1999), with minor changes. The apparatus consisted of a plexiglass pool (30 cm × 100 cm × 8 cm) filled with water (23°C). It has an escape platform in the opposite side of the one the animal is placed in to start the test. During the test the mouse must reach the platform swimming from the opposite side, the latency time is recorded meanwhile. Before the test the mice were trained among few trials in which the platform started close to the initial place of the mouse and was put farther in each trial.

### Histology

#### Tissue preparation

After intraperitoneal pentobarbital (intraperitoneal 400 mg/mL) overdose, mice were perfused transcardially with phosphate buffer (0.1 M) followed by 4% paraformaldehyde (PFA). Brains were post-fixed in PFA. Once fixed, the brains were transferred to 30% (w/v) sucrose for dehydration and cryoprotection for at least 48 h, after which they were frozen using isopentane cooled with dry ice at −80°C. They were immersed in the isopentane for some seconds (around 30 s) and them wrapped and stored at −80°C. Coronal 30 μm sections were obtained with a sliding microtome (SM2010R, Leica) and stored in cryoprotective solution.

#### Nissl staining and hippocampal volume measurements

For hippocampal volume measurements, 30-μm sections taken every 300 μm were stained with cresyl violet (Sigma-Aldrich). Stained sections were visualized under a light microscope (Nikon Eclipse 80i, Nikon Corporation) (10X lens). The volume of the hippocampus and the DG were estimated stereologically by applying the Cavalieri method, performed with Stereo Investigator software (MicroBrightField), for each series of Nissl-stained sections. Volumes were estimated by using a 1-in-5 systematic random series of 30 μm Nissl-stained sections using a 100 and 20 μm^2^ point-counting grid, respectively.

#### Immunohistochemistry

Immunohistochemistry (IHQ) was performed using an automatic IHQ system. First step was an antigen retrieval consisting of a 30-min incubation at 37°C in 10 mM Tris 1 mM EDTA solution. Next, slices were blocked in 0.3% Triton X-100 and 1% bovine serum albumin (BSA) in PBS for 1 h and incubated with primary antibody in the same solution over night at 4°C. The primary antibody was anti-caspase-3 (Asp175) (1:100, Cell signaling). After rinsing, the slices were incubated with the secondary antibody (HRP anti-rabbit) and revealed with diaminobenzidine. Hematoxylin staining was also performed (Alonso-Alconada et al., 2022).

#### Immunofluorescence

Immunofluorescence was performed on free-floating sections. Briefly, sections were first permeabilized and blocked in 0.3% Triton X-100 in PBS with 10% normal serum for 2 h or 1% BSA and then incubated overnight at 4°C in 0.3% Triton X-100 in PBS with 5% normal serum or 1% BSA and the following primary antibodies: anti-DCX (neuroblast marker, 1:400 Abcam) and anti-Ki-67 (proliferation marker, 1:200, Invitrogen).

The secondary antibodies used were biotinylated goat anti-rabbit (1:400, VectorLab), streptavidin-488 (1:500, Biolegend), and goat anti-rat 568 (1:500, Thermo Fisher).

#### Brain-blood barrier (BBB) integrity

Anti-IgG fluorescence staining was performed to measure BBB integrity. The immunofluorescence was performed as previously described by using the secondary antibody biotinylated goat anti-mouse (1:500 VectorLab) and followed by 488-streptavidin (1:400) staining. Measurement of IgG extravasation was performed under a fluorescence microscope (Nikon Eclipse 80i, Nikon Corporation). IgG^+^ regions were traced manually and volumetric extravasation measurements were performed using the cavaliery estimation of the software StereoInvestigator 6 (MicroBrightfield Inc.).

#### Image processing and analysis of immuno-stained sections

Image acquisition was performed with a laser-scanning confocal imaging system (Zeiss LSM710, 25X, and Leica TCS SP8, 40X) and image quantification was performed with ImageJ software (NIH), and Imaris (Oxford Instruments). DCX + cells and DCX, Ki-67 co-immunostaining were manually quantified in confocal z-stack images (25X lens and 40X lens, respectively) by the 3D reconstruction in Imaris. Specifically, confocal stacks of images of both hemispheres’ dentate gyri were obtained from 3 to 4 rostral sections. Cells were counted manually, and data were expressed as number of cells per section. For neuroblast morphology, high-resolution confocal images stacks of hippocampus from 3 rostral sections were obtained. At least 30 neurons were traced and analyzed using Imaris filaments tool. Apical dendrite length was measured in Imaris.

### ASL-MRI

For the image acquisition, mice were deeply sedated using isoflurane al 2% and 1.8 L/min oxygen flow. Ophthalmic gel was placed in their eyes to prevent retinal drying. Body temperature was maintained at 37°C to avoid hypothermia using a hot air flow system controlled by a rectal probe which measures animal temperature (SAII gating and monitoring systems for small animal, SA Instruments).

Cerebral blood flow (CBF) was carried out using an Arterial Spin Labeling (ASL) based method which consists on a flow-sensitive alternating inversion recovery magnetic resonance imaging (MRI) technique (Kober et al., 2004; Schindelin et al., 2012). ASL was performed by using a 7-T Agilent/Varian scanner (Agilent, Santa Clara, CA, USA) equipped with a DD2 console and an actively shielded 205/120 gradient and a combination of a volume and surface coil for MR signal transmission and reception.

Arterial spin labeling images of an axial slice [hippocampus level (bregma level −2 mm)] were acquired in a non-selective and slice-selective experiments. For each experiment, twenty-two images with different inversion times were acquired using fast spin multislice (fsems) sequence with the following parameters: TR/TE, 10000/5.05 ms; number of averages, 1; matrix size, 96 × 96; FOV, 23 mm × 20 mm; in-plane spatial resolution, 240 μm × 208 μm; slice thickness, 2 mm; and number of slices, 1. The following inversion time values were used: 30, 100, 200, 300, 400, 500, 600, 700, 800, 900, 1000, 1100, 1200, 1300, 1400, 1500, 1600, 1700, 1800, 1950, 2100, and 2300 ms. Total scan time was 22 min 20 s. The CBF image was calculated from the obtained 44 images using MatLab (MathWorks) following the equations described (Schindelin et al., 2012). Region of interest (ROI) analyses of CBF images were carried out using the free software FIJI (Schindelin et al., 2012).

### DTI-MRI

For *ex vivo* diffusion tensor imaging (DTI) studies, mice were perfused transcardially with 0.1 M phosphate buffer (pH 7.4) followed by 4% paraformaldehyde (PFA; pH 7.4). Brains were removed and preserved in PFA. DTI experiments were carried out on a Bruker Biospec BMT 47/40 (Bruker BioSpin) operating at 4.7 T and equipped with a 6.0-cm gradient system. Before the imaging experiments, the PFA excess of the sample was eliminated, and it was introduced inside and the appropriate holder filled with Fluorinert FC 40 (CAS number 51142-49-5; Milli-pore Sigma, Burlington, MA, USA) and then placed inside a 3.5-cm birdcage radiofrequency coil. For the DTI images, a spin-echo sequence (TR/TE = 3500/30 ms) was used. A total of 14 different images for each slice was acquired, 2 of them without diffusion weighting and 12 different directions of the diffusion gradients, all of them with a b-factor of 1000 s/mm^2^. The fractional anisotropy maps were calculated using the software ParaVision 6.0.1 (Bruker BioSpin). The brain structures segmentation and fractional anisotropy quantification from these maps were carried out using the free software ImageJ 1.50i (National Institutes of Health, Bethesda, MD, USA).

### Statistical analysis

The results were expressed as the mean ± S.E.M. Statistical analyses were conducted using Prism9 software (GraphPad Software, Inc., USA). Given that the sample size (n) hindered to assess normality and homoscedasticity, non-parametric methods were employed. This approach allowed us to avoid making any *a priori* assumptions about the distribution of the data. Differences between two groups were tested using a two-tailed Mann-Whitney *U*-test. In experiments with a factorial design, factors were analyzed using a 2-way ANOVA, followed by multiple Mann-Whitney post-tests with Holm-Sidak correction. Statistical significance was considered if *p* < 0.05.

## Results

### BCAS impairs hippocampal spatial memory

Consistent with previous studies (Shibata et al., 2007; Füchtemeier et al., 2015; Hattori et al., 2016; Boehm-Sturm et al., 2017; Koizumi et al., 2018; Liu et al., 2019), our BCAS model significantly reduced CBF in both hippocampus and cortex 1 and 3 months after surgery (Figure 1B), resulting in a trend toward white matter connectivity impairment in the anterior commissure at 3 months (Supplementary Table 1 and Supplementary Figure 3). In addition, blood-brain barrier (BBB) leakage was detected by IgG extravasation in the hippocampus (Supplementary Figure 1).

While no differences in depression or anxiety like behavior (Supplementary Figure 2) were detected, hypoperfused mice displayed deficits in spatial working memory, assessed by the Y-maze spontaneous alternation performance (SAP) task, only after 3 months of stenosis (Figure 1C). In the novel object location (NOL) test at 1 and 3 months, BCAS mice displayed a recognition index lower than sham mice at 3 months, indicating that chronic hypoperfusion promotes a failure also in hippocampal spatial reference memory skills (Figure 1D).

### BCAS-induced hippocampus-dependent memory deficits are concomitant to impaired hippocampal neurogenesis in numbers and morphology

**FIGURE 2:**
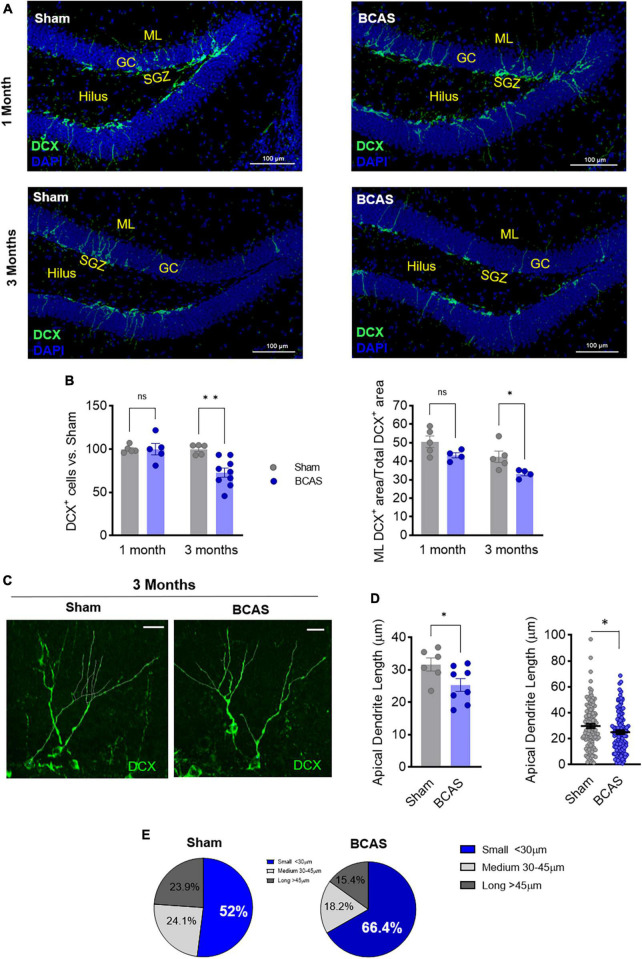
Memory deficits are concomitant to impaired hippocampal neurogenesis following hypoperfusion. **(A)** Representative images of doublecortin (DCX) staining in the DG. Scale bar = 100 μm. **(B)** DCX^+^ cells number (left) and % of DCX^+^ area in the molecular layer (ML; right) after BCAS (*n* = 4–9). **(C)** Representative high-resolution images of DCX^+^ cells. Scale bar = 15 μm. **(D)** Quantification of apical dendrite length. Left: mean apical dendrite by animal; right: distribution of every apical dendrite length (*n* = 6–8). **(E)** Pie charts of the distribution in apical dendrite length. Statistical assessment by ANOVA and multiple Mann-Whitney tests with Holm-Sidak correction for the paired comparisons. Two comparisons were tested by Mann-Whitney *U*-test. *p < 0.05, **p < 0.01.

Since BCAS-induced chronic cerebral hypoperfusion impairs hippocampal-dependent memory, we focused on the neurological substrates involved in these deficits. By Nissl staining evaluation, we did not observe any major histological or volumetric hippocampal differences between sham and BCAS mice (Figure 1E). We next explored possible alterations in hippocampal neurogenesis, proposed to underlie hippocampal cognitive impairment in different scenarios (Kim et al., 2022). Of note, in association with impaired hippocampus-dependent memory tasks, the data showed strong evidence that hypoperfusion has a negative effect on the number of immature neurons (neuroblasts; DCX^+^) (median contrast: −30.17, 29.03% reduction; 95.8% CI: −45.69 to −10.84; Cohen’s *d* = 2.257) in the DG compared to sham group at 3 months after stenosis (Figures 2A, B). As the capability to form and store new memories not only relies on the number of neuroblasts but also on their arborization and ability to integrate into hippocampal circuits, we investigated the dendritic arbor of the newborn DCX^+^ neurons. Importantly, we found a reduction in the DCX^+^ area located in the molecular layer relative to the total positive DCX^+^ area in BCAS-operated mice (Figures 2A, B), suggesting morphological changes in the arborization of neuroblasts after hypoperfusion. To further explore this altered neuroblast morphology, apical dendrite length was measured in high resolution images with a 3D image. The analysis showed evidence on the shortening (median contrast: −6.47 μm, 19.99% reduction; 95.74% CI: −13.39 to −0.11; Cohen’s *d* = 1.195) of the mean apical dendrite length in hypoperfused mice (Figures 2C, D) vs. sham group at 3 months, with an increase in the percentage of immature neurons with shortened apical dendrite length (Figure 2E).

Therefore, our data support that 3-months BCAS induces a decrease in the number of neuroblasts and an increase in the number of aberrant newborn neurons with shortened apical dendrite, pointing to both quantitative and qualitative failures in newborn neurons integration into hippocampal circuits, which may underlie spatial cognitive dysfunction in BCAS mice.

### Apoptosis and decreased neuroblast proliferation rate are associated with BCAS-induced impaired hippocampal neurogenesis

**FIGURE 3:**
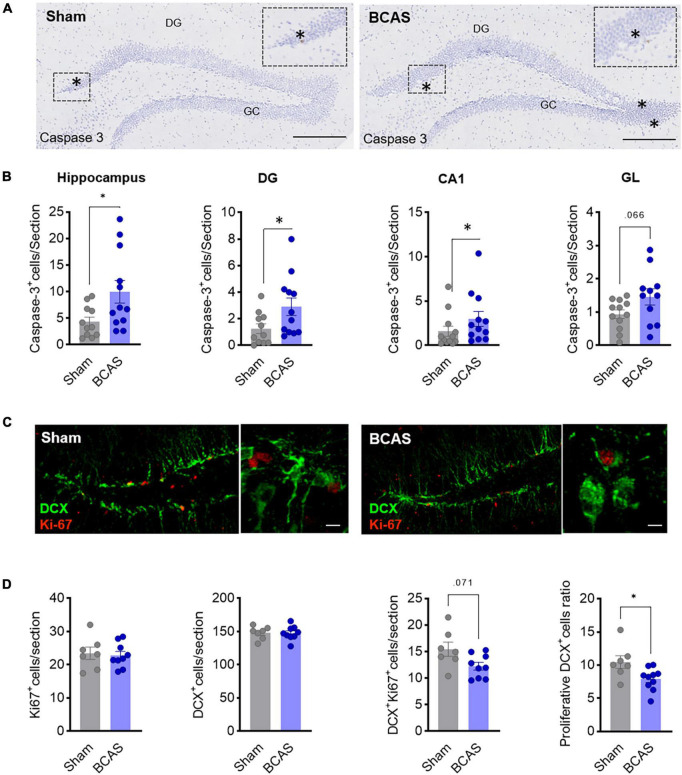
Mechanisms leading to impaired hippocampal neurogenesis. **(A)** Representative images of cleaved caspase-3 in hippocampus 3 months after surgery. Scale bar = 250 and 50 μm. **(B)** Cell death quantification in different hippocampal regions. Mann-Whitney test was used to compare the ranks (*n* = 12). **(C)** Representative images of DCX (green) and Ki-67 (red) co-staining in DG 1 month after surgery. Scale bars = 80 and 15 μm (*n* = 7–9). **(D)** Quantification of Ki67^+^, DCX^+^, and Ki67^+^/DCX^+^ cells and of the proliferation ratio of DCX^+^ cells. Two-tailed Mann-Whitney *U*-test was used to compare the ranks. *p < 0.05.

As a potential mechanism to explain the reduced number of newborn neurons in BCAS mice, we first evaluated whether hypoperfusion was affecting cell survival. Indeed, BCAS mice presented higher numbers of activated nuclear caspase-3^+^ cells vs. sham 3 months after BCAS in total hippocampus (including CA1) and DG (median contrast: 1.431, 186,06% increment; 95.61% CI: 0.1474 to 3.446; Cohen’s *d* = −0.906), and a trend toward an increase in GL (Figures 3A, B), demonstrating BCAS-increased apoptotic cell death in areas where neurogenesis takes place and suggesting that reduced cell survival might account for decreased neuroblasts numbers after BCAS.

In addition, we also investigated whether proliferation rate was affected by analyzing Ki67 cells 1 month after stenosis. Remarkably, although no differences in the total number of Ki67^+^ and DCX^+^ cells in the DG were found, we observed evidence about the reduction in the ratio of DCX^+^-Ki67^+^/DCX^+^ in the BCAS group (median contrast: −1.708, 17.03% reduction; 95.69% CI: −5.061 to −0.333; Cohen’s *d* = 1.197) and a trend toward a reduction in the number of proliferative DCX cells (median contrast: −2.962, 19.8% reduction; 95.82% CI: −6.185 to 0.064; Cohen’s *d* = 1.09) (Figures 3C, D), strongly suggesting that hypoperfusion promotes a decrease in neuroblast proliferation rate which may explain, together with increased apoptosis, the decay in neurogenesis in the BCAS mice along time.

Altogether, these data support the notion that both increased apoptosis and reduced proliferation rate account for quantitatively altered neurogenesis in BCAS mice.

## Discussion

Due to the heterogeneity of the mechanisms underlying cognitive deficits and the scarcity of effective animal models to recapitulate them, the pathophysiological knowledge and the development of treatments for VCID remain an urging therapeutic and diagnostic challenge. Specifically, since chronic brain hypoperfusion has been associated with VCID in human patients (Iadecola, 2013), a murine model of bilateral carotid stenosis was developed (Shibata et al., 2004) that is considered to recapitulate subcortical white matter dementia by cerebral small vessel disease (Füchtemeier et al., 2015; Hainsworth et al., 2017; Ohtomo and Arai, 2020). This BCAS model has been traditionally associated with working memory deficits consistent with white matter damage (Shibata et al., 2007; Nishio et al., 2010; Hainsworth et al., 2017). Now, we demonstrate that BCAS-induced global cerebral hypoperfusion also induces hippocampus-dependent spatial reference memory deficits concomitant to a significant quantitative and qualitative impairment in SGZ neurogenesis.

First of all, we characterized our model compared to the data in the literature as regards the extent, nature, features and lesions presented. Our results show CBF reductions in good agreement with the data in the literature (Shibata et al., 2004; Hattori et al., 2016), with CBF percentages in the long-term near 80% of sham-operated or pre-surgery CBF. As regards behavioral data, our 3-month-BCAS model presented neither anxiety, motor disturbances, hyperlocomotion nor behavioral despair as previously described at 2 months after surgery (Shibata et al., 2007; Nishio et al., 2010). When exploring cognition, we did not find working memory impairment in the Y-maze until 3 months after BCAS, compared to 2–4 weeks in previous studies (Dong et al., 2011; Maki et al., 2011). Overall, these data corroborate that our BCAS model reproduces, although to a somewhat milder extent, most of the phenotypic features previously reported. As regards white matter damage (WMD), our BCAS model seems to reproduce data in the literature: when we assessed the fractional anisotropy (FA) by DTI-MRI to explore axonal organization we found a significant disorganization in the anterior commissure of BCAS-operated mice. Consistently, several reports described WMD by different histological measurements (Shibata et al., 2007). In this context, some studies failed to observed differences in FA 2 months after surgery but they did observe signs of axonal connectome impairment in several DTI parameters (Boehm-Sturm et al., 2017).

However, alterations in gray matter structures and specifically of hippocampus were quite underexplored. In this context, we found variable IgG extravasation in hippocampus of the BCAS group, suggesting a disruption of the BBB in this area, only reported previously in corpus callosum and cortex (Nakaji et al., 2006; Liu et al., 2019). We therefore decided to investigate the NOL memory, which relies primarily on hippocampal neural processes. Notably, our model showed a clear impairment in the NOL task, strongly supporting a hippocampal involvement. This is in contrast with previous data in the literature which reports deficits in hippocampus-dependent spatial reference memory only after 5–6 months of microcoil implantation (Shibata et al., 2007; Nishio et al., 2010; Coltman et al., 2011).

Several studies on post-stroke cognitive impairment, including ours, support that spatial memory deficits are associated with alterations in adult hippocampal neurogenesis, a process taking place in the mature brain SGZ (Cuartero et al., 2019, 2021). Indeed, NOL deficits were concomitant with a remarkable impairment of the SGZ neurogenesis, displayed both as a reduced number of newborn neurons as well as an increased number of immature neurons displaying an aberrant morphology, supporting that BCAS-induced cerebral hypoperfusion results in a qualitative and quantitatively defective integration of newborn granule neurons into the hippocampal circuits, subsequently leading to an impaired hippocampus-dependent memory. To our knowledge, few studies have investigated neurogenesis status in the BCAS context. Two reports described some trend toward a decrease in the number of proliferative or DCX^+^ cells in the SGZ 6 weeks after BCAS surgery (Higaki et al., 2018; Song et al., 2018) and one report, using the more aggressive bilateral common carotid artery occlusion (BCCAO) model, found a remarkable decrease in DCX + cells as early as 14 days after BCCAO (Soares et al., 2013). In our model, hypoperfusion decreased the number of immature neurons in a 30%, a reduction lower than that induced in the severe BCCAO model (50% descent) (Higaki et al., 2018), suggesting that it is a response which depends on the extent of the hypoperfusion. Interestingly, our result is quite comparable to that observed in Braak I stage Alzheimer’s human patients (33% reduction) (Moreno-Jiménez et al., 2019).

We finally explored several potential mechanisms underlying defective neurogenesis in BCAS model. The neurogenic cascade is a highly regulated process at different steps including neural stem cell maintenance, proliferation, fate specification and differentiation, migration, full maturation and, finally, newborn neuron integration into the local circuitry. Then, we reasoned that the reduction in the number of newborn granule neurons could be due to increased cell death and/or decreased proliferation rate. Our results suggest that both mechanisms could be involved: on the one hand, the number of nuclear cleaved-caspase-3^+^ cells in the DG was increased after BCAS; on the other hand, we found an early reduction in the proliferation ratio of neuroblasts after 1 month of BCAS that could result in a reduced number of neuroblasts over time. As regards the qualitative alterations in neuroblasts morphology, we found a 19% shorter apical dendrite length in the BCAS compared to the sham animals. This observation is particularly significant in the context of the hippocampal circuit, as the proper arborization and connectivity of granule neurons with afferent pathways from the entorhinal cortex (EC) have been described to be essential for hippocampal function (Shapiro et al., 2007). Previous studies have described alterations in dendritic arborization, including shortening of the apical dendrite, in various pathological conditions, such as Alzheimer’s disease and stroke (Llorens-Martín et al., 2015; Cuartero et al., 2021). These changes have been associated with impaired plasticity and failure of hippocampal connectivity (de la Parra et al., 2018; Márquez-Valadez et al., 2022). The findings in our study are consistent with these previous reports, highlighting the potential implications of reduced apical dendrite length on the plasticity and connectivity of the hippocampal circuitry. Although further research is needed to clarify how these mechanisms take place longitudinally and their contribution, all these data pose AHN as a common theme in different settings of dementia, including in chronic stroke (Cuartero et al., 2019).

## Data availability statement

The raw data supporting the conclusions of this article will be made available by the authors, upon reasonable request.

## Ethics statement

The animal study was reviewed and approved by the Animal Welfare Committee of the Centro Nacional de Investigaciones Cardiovasculares Carlos III (CNIC) and the Consejería de Medio Ambiente y Ordenación del Territorio de la Comunidad de Madrid (RD 53/2013; PROEX 047/16, PROEX 193.1/21).

## Author contributions

EF, VM, AG-C, MC, MH-J, JG-S, IL, and MM designed the research studies. EF, VM, AG-C, MC, and IB-F conducted the experiments and/or acquired the data and wrote the manuscript. EF, VM, MC, AG-C, JG-S, OH, JMP, IL, and MM contributed to the analysis and/or interpretation of the results. All authors reviewed and approved the manuscript.
