# Supplementary material for: Defective hippocampal neurogenesis underlies cognitive impairment by carotid stenosis-induced cerebral hypoperfusion in mice
Source: Front Cell Neurosci. 2023 Aug 11;17:1219847. doi: 10.3389/fncel.2023.1219847 (PMC10457159; doi:10.3389/fncel.2023.1219847)
Supplement: Supplementary file 1 [file Data_Sheet_1.docx]

**DATA SUPPLEMENT**

**Supplementary Table 1**

| ***Region*** |  | |  | | ***1-month*** | |  | | ***3-month*** | | |  |
| --- | --- | --- | --- | --- | --- | --- | --- | --- | --- | --- | --- | --- |
|  | **Sham**  Median | **BCAS**  Median | | **Contrast** | | **86.67% CI** | **Sham**  Median | **BCAS**  Median | | **Contrast** | **66.67% CI** | |
| *Hippocampus* | 0.327 | 0.329 | | -0.001875 | | -0,06750 to 0,03900 | 0.300 | 0.282 | | -0.01808 | -0,04267 to 0,006500 | |
| *Anterior Commissure* | 0.522 | 0.488 | | -0,03449 | | -0,09337 to 0,03360 | 0.543 | 0.367 | | -0,1756 | -0,1987 to -0,1525 | |
| *Internal Capsule* | 0.441 | 0.432 | | -0,008708 | | -0,06350 to 0,01667 | 0.475 | 0.416 | | -0,05867 | -0,08550 to -0,03183 | |
| *Striatum* | 0.305 | 0.296 | | -0,009083 | | -0,05217 to 0,03033 | 0.288 | 0.288 | | 0,0002500 | -0,02550 to 0,02600 | |
| *Corpus Callosum* | 0.496 | 0.471 | | -0.02567 | | -0,05633 to 0,008333 | 0.471 | 0.460 | | -0.01067 | -0,02667 to 0,005333 | |
| *Fornix* | 0.493 | 0.456 | | -0,03750 | | -0,09700 to 0,03000 | 0.430 | 0.465 | | 0,03450 | 0,006000 to 0,06300 | |
| *Thalamus* | 0.336 | 0.311 | | -0,02525 | | -0,05500 to 0,03475 | 0.290 | 0.276 | | -0,01425 | -0,05000 to 0,02150 | |
| *Cortex* | 0.312 | 0.294 | | -0,01825 | | -0,05130 to 0,004600 | 0.266 | 0.283 | | 0,01741 | -0,02280 to 0,05763 | |
| *Prefrontal cortex* | 0.328 | 0.310 | | -0,01775 | | -0,05609 to 0,01057 | 0.029 | 0.314 | | 0,02255 | -0,002438 to 0,04755 | |

**Supplementary Table 2**

| Testing | Initial sample size | Final sample size  (without outliers) |
| --- | --- | --- |
| *ASL* | Sham: 8  BCAS: 16 | Sham: 8  BCAS: 16 |
| *Y maze 1 month* | Sham: 7  BCAS: 7 | Sham: 7  BCAS: 7 |
| *Y maze 3 months* | 5 | 5 |
| *NOL 1 month* | Sham: 7  BCAS: 5 | Sham: 6  BCAS: 5 |
| *NOL 3 months* | Sham: 7  BCAS:5 | Sham: 6  BCAS:5 |
| *Nissl 1 month* | 5 | 5 |
| *Nissl 3 months* | 4 | 4 |
| *DCX^+^ cells 1 month* | 5 | 5 |
| *DCX^+^ cells 3 months* | Sham: 5  BCAS: 9 | Sham: 5  BCAS: 9 |
| *DCX^+^ cells 1 month* | 5 | 5 |
| *DCX^+^ cells 3 months* | 4 | 4 |
| *DCX morphology* | Sham: 6  BCAS: 8 | Sham: 6  BCAS: 8 |
| *Caspase-3* | 12 | Sham:10-12  BCAS:11-12 |
| *DCX^+^ Ki-67^+^ cells* | Sham:7  BCAS: 10 | Sham:7  BCAS: 9 |
| *DTI 1 month* | Sham: 2  BCAS: 4 | Sham: 2  BCAS: 4 |
| *DTI 3 months* | 2 | 2 |
| *IgG* | Sham: 6  BCAS 1 month: 10  BCAS 3 months: 6 | Sham: 5  BCAS 1 month: 10  BCAS 3 months: 6 |
| *FST 1 month* | Sham: 7  BCAS: 6 | Sham: 7  BCAS: 6 |
| *FST 3 months* | 6 | 6 |
| *MTS 1month* | Sham: 4  BCAS: 5 | Sham: 4  BCAS: 5 |
| *MTS 3 months* | 8 | 8 |
| *OF* | Sham: 8  BCAS:7 | Sham: 8  BCAS:7 |

**Supplementary Tables legends**

**Suppl. table 1. Effects of 1 and 3 months of hypoperfusion in the white matter organization.** Fractional anisotropy was measured by *ex vivo* MRI in the hippocampus, the anterior commissure, the internal capsule the striatum, the corpus callosum, the fornix, the fimbria the thalamus the prefrontal cortex and the cortex. ANOVA was performed followed by multiple Mann-Whitney tests with Holm-Sidak correction for the paired comparisons; (n = 2-4).

**Suppl. table 2. Sample size by experiment.** The detailed sample sizes of each experiment initially and after excluding any outlier are shown.

**Supplementary Figures legends**

**Suppl. fig. 1. Hippocampal blood brain barrier is disrupted after 3 months of hypoperfusion.** An anti-endogenous-murine-IgG immunofluorescence was performed and the extravasation of IgG measured in the hippocampus. **(A)** Representative images of IgG staining (IgG in green). **(B)** IgG extravasation measurement by IgG^+^ volume (mm^3^). (C) Contingency table representation. (D) Boxplot graph of positive IgG volume. χ^2^ was employed to analyse the contingency table; n = 6-10.

**Suppl. fig. 2. Behavioral testing in the BCAS model. (A)** Forced swimming test was performed after 1 and 3 months of hypoperfusion to evaluate the depressive behavior of the mice. **(B)** Motor swimming test was also performed after 1 and 3 months after BCAS to test the locomotor activity. **(C, D)** Open field test was used to explore the anxiety of the mice. **(C)** Ratio between the time expend in the periphery and the center of the arena. **(D)** Travelled distance along the arena. ANOVA was performed and followed by multiple Mann-Whitney tests with Holm-Sidak correction for the paired comparisons; n = 8.

**Suppl. fig. 3. Fractional anisotropy (FA) images.** Representative images of the FA assessment in 2 different sections from one sham and one BCAS animal. Anatomical images are showed alongside the FA images.

**Suppl. fig. 4. Graphical design and distribution of the cohort of animals used for each behavioral testing.** Visual representation of the allocation of the groups of animals for each behavioral test. (A) 1-month groups. (B) 3-month groups.

**Supplementary File descriptions**

**Suppl. File 1. Statistical power calculation of the statistical tests performed for the experiments.** Console R output showing the statistical power achieved for each test using an alpha=0.05 and the means and SD from the results. The function pwrss.np.2groups() from the package pwrss was used.

**Suppl. File 2. R code to perform the statistical power calculation.** The file is Suppl_File_2.R and contains the code used to analyze the statistical power in Suppl. File 1.
