# Supplementary figures and images for: Defective hippocampal neurogenesis underlies cognitive impairment by carotid stenosis-induced cerebral hypoperfusion in mice
Source: Front Cell Neurosci. 2023 Aug 11;17:1219847. doi: 10.3389/fncel.2023.1219847 (PMC10457159; doi:10.3389/fncel.2023.1219847)

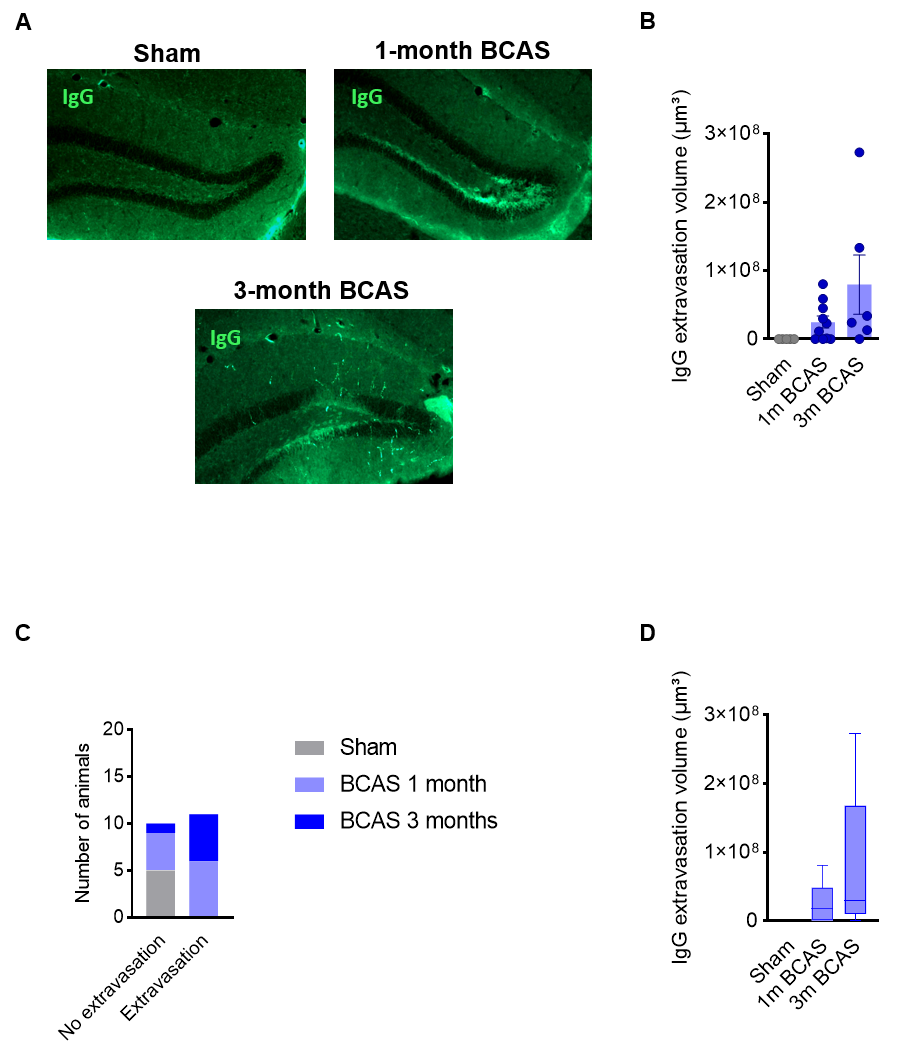

Supplement: Supplementary file 2 [file Data_Sheet_2.zip › Suppl Fig 1.tif]

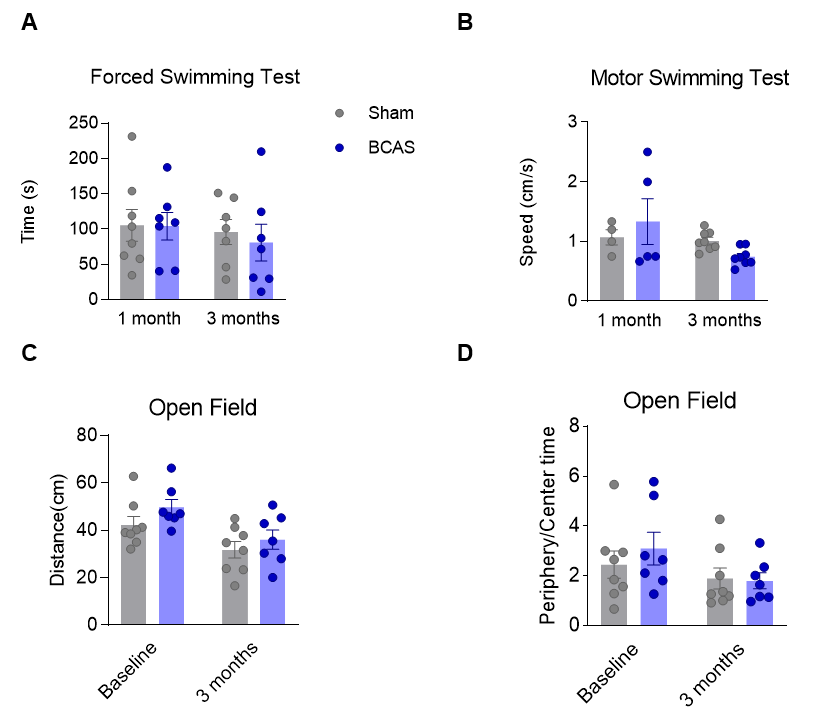

Supplement: Supplementary file 2 [file Data_Sheet_2.zip › Suppl Fig 2.tif]

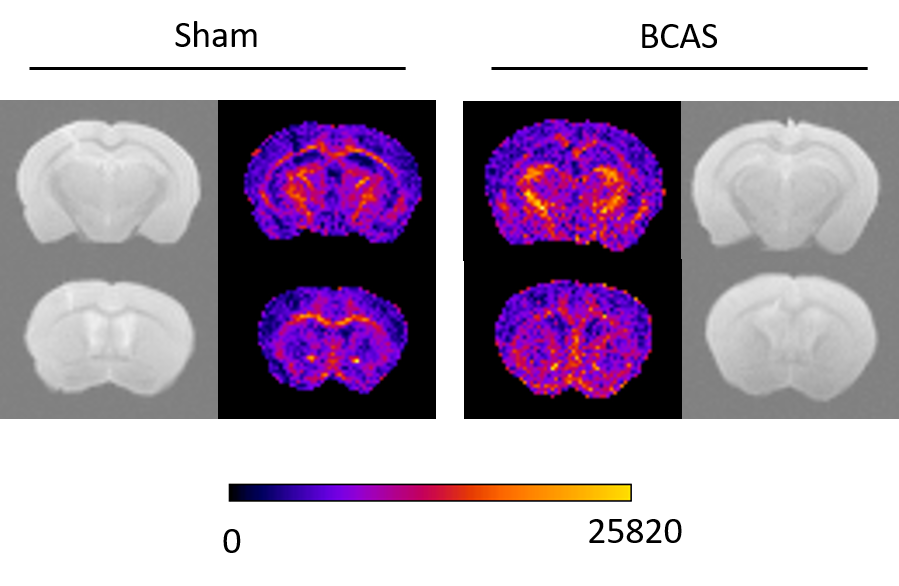

Supplement: Supplementary file 2 [file Data_Sheet_2.zip › Suppl Fig 3.tif]

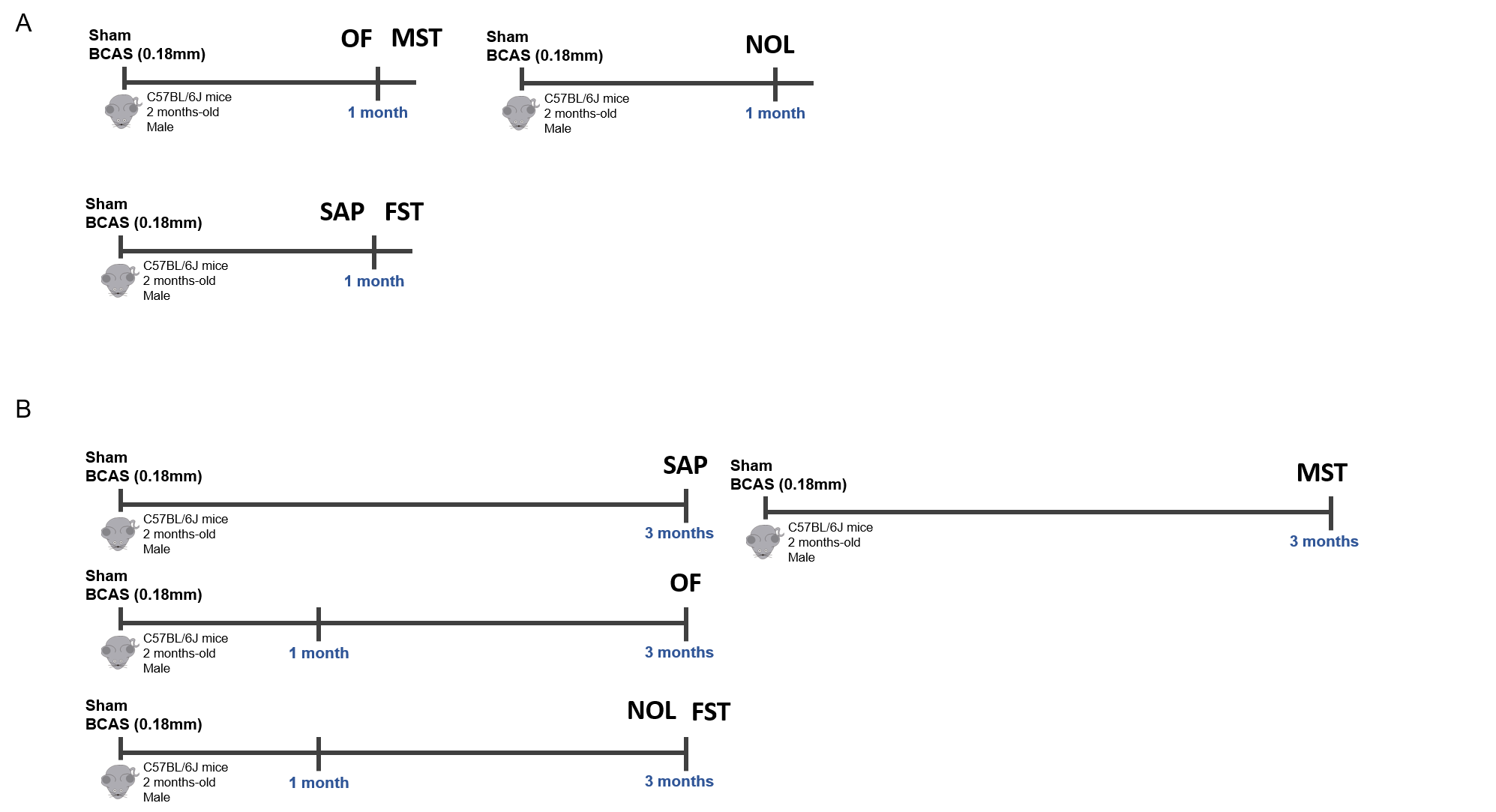

Supplement: Supplementary file 2 [file Data_Sheet_2.zip › Suppl Fig 4.tif]
